# Phytochemical Characterization and Utilization of Dried Red Beetroot (*Beta vulgaris*) Peel Extract in Maintaining the Quality of Nile *Tilapia* Fish Fillet

**DOI:** 10.3390/antiox11050906

**Published:** 2022-05-05

**Authors:** Hossam S. El-Beltagi, Mohamed M. El-Mogy, Aditya Parmar, Abdallah Tageldein Mansour, Tarek A. Shalaby, Marwa Rashad Ali

**Affiliations:** 1 Agricultural Biotechnology Department, College of Agriculture and Food Sciences, King Faisal University, P.O. Box 420, Al Hofuf 31982, Saudi Arabia; 2 Biochemistry Department, Faculty of Agriculture, Cairo University, Gamma St, Giza 12613, Egypt; 3 Vegetable Crops Department, Faculty of Agriculture, Cairo University, Giza 12613, Egypt; elmogy@agr.cu.edu.eg; 4 Natural Resources Institute, University of Greenwich, Central Avenue, Chatham Maritime, Kent ME4 4TB, UK; a.parmar@gre.ac.uk; 5 Animal and Fish Production Department, College of Agriculture and Food Sciences, King Faisal University, P.O. Box 420, Al Hofuf 31982, Saudi Arabia; amansour@kfu.edu.sa; 6 Fish and Animal Production Department, Faculty of Agriculture (Saba Basha), Alexandria University, Alexandria 21531, Egypt; 7 Department of Arid Land Agriculture, College of Agricultural and Food Science, King Faisal University, P.O. Box 400, Al Hofuf 31982, Saudi Arabia; tshalaby@kfu.edu.sa; 8 Horticulture Department, Faculty of Agriculture, Kafrelsheikh University, Kafr El-Sheikh 33516, Egypt; 9 Department of Food Science, Faculty of Agriculture, Cairo University, Giza 12613, Egypt

**Keywords:** beetroot peel, phenolic compounds, IC 50%, fish fillet, TBA

## Abstract

Phytochemicals derived from agro-industrial waste materials could be employed as functional food additives and natural antioxidants to replace their synthetic counterparts, which are increasingly being rejected. The current study aims to assess total phenolic compound (TPC), flavonoids, betalain contents, and antiradical scavenging using DPPH and IC50% of dried red beetroot peel (DRBP) extract at different concentrations of 50, 80, 100, 150, and 200 mg/100 mL t. In addition, a characterization of phenols and flavonoids was conducted using HPLC. The second part of this study aims to utilize aqueous DRBP extract in preserving Nile *Talipia* fish fillet at two concentrations of 80 and 100 mg/100 mL water, compared with 200 ppm of BHT (butylated hydroxytoluene) and control at 5 °C for 10 days. The DRBP aqueous extract was found to have a high concentration of TPC (832 mg/100 g), flavonoids (234 mg/100 g) and betalains (535 mg/100 g) compounds, resulting in a potential antioxidant activity. The IC50% for the extract was detected at 80 mg/100 mL extract. DRBP aqueous extract showed an excellent preservative effect on the fish fillet. Fish fillet samples treated with DRBP extract at a concentration of 100 mg/100 mL were superior in reducing TBA (thiobarbituric acid) increase compared with other treatments at the end of cold storage. Overall, the study showed that red beetroot extracts can act as a natural preservative agent due to their significant antioxidant activity, providing healthy and safe food to consumers.

## 1. Introduction

Nile *Tilapia* (*Oreochromis niloticus*) is one of Egypt’s most valuable economic marine fish, preferred for its good nutritional content and superior sensory attributes. The consumption of this fish in Egypt increased from 8.5 to −15.4 kg/person/year between 1996 and 2008. Egypt is the largest producer of farmed *Tilapia* fish in Africa and the second internationally after China [1]. Customers in Egypt consider fresh seafood a superior commodity to its frozen alternative [2]. Due to its biological nature, fresh seafood is highly perishable and has a short shelf-life. Fish spoilage normally occurs through lipid oxidation, autolytic enzymes, and microorganisms’ metabolic activities [3].

Application of refrigeration can help slower the fish spoilage process, however, over time metabolic and microorganisms activity result in shorter shelf-life and poorer safety and quality. This poses a high risk to consumer health and economic loss to the producers. Lipid oxidation in fish products may deteriorate the lipids and the color impacting the visual acceptability of consumers. Utilization of natural preservatives in improving the shelf-life and food safety of fish products has a significant potential, which has not been fully realized [4].

Synthetic antioxidants such as butylated hydroxytoluene (BHT), and butylated hydroxyanisole (BHA), are commonly used in the food industry to prevent the negative effects of free radicals. The Food and Drug Administration (FDA) restricted the use of synthetic antioxidants due to their toxicity and carcinogenic potential [5]. As a result, powerful antioxidants from natural sources are required for such applications. Many studies have shown that natural substances and numerous crude extracts exhibit antioxidant and radical-scavenging properties [6]. High antioxidant activity has been associated with phenolic components such as phenolic acids and flavonoids [7]. Recently, the marine industry and academics have taken an interest in utilizing preservatives such as essential oils and phosphates as an alternative to artificial chemicals. They benefit from being approved by consumers and can inhibit microbial growth, improve health, reduce lipid oxidation, and minimize drip and cooking losses, thus improving the safety, acceptability and shelf-life of chilled fish products [8]. A previous study on salmon fish revealed that the addition of natural antioxidants in edible films enhanced the shelf-life and preserved the samples from oxidation during frozen storage [9]. It has been reported that thyme extract has powerful antimicrobial and antioxidant activities and can retain the quality and prolong the shelf-life of chilled Nile *Tilapia* fillets for 9 days longer than the control [10]. In the same study, rosemary extract showed a strong antioxidant activity with slightly lower antimicrobial action.

Beetroot (*Beta vulgaris* L.) belonging to the *Chenopodiaceae* family, are of various colors varying from yellow to red. Dark-red-colored beetroots are generally eaten in raw or cooked forms. Red beetroot is one of the most potent vegetables for antioxidant activity due to several active compounds such as carotenoids, glycine betaines, saponins, and folates of beta-cyanlins and betalains polyphenols, and flavonoids [11]. According to FAO statistics in 2018, the global beetroot production was about 274 million tonnes. The majority of red beetroot (85%) is used for processing and about 30% of the total production was lost or wasted during processing due to poor root quality [12]. Reusing red beetroot wastes resulting from one processing operation could add value to the supply chain [13].

In view of the circular economy and reducing food wastes, side streams and by-products such as peels and seeds collected from fruit and vegetable processing plants could be a potential source of phytochemicals and antioxidants (particularly phenolic compounds) [14]. In certain cases, by-products such as fruits and vegetable peels could represent up to 40% of the total weight and contribute to significant pollution if not properly handled [15]. Therefore, the recovery of plant by-products, including refining them into economically innovative value-added products is an important aspect towards more sustainable food supply chains [16].

Beetroot peel has antioxidant compounds, which indicate its usefulness in the food and nutritional supplements industries. Compared with other vegetable peel extracts, beetroot extracts, particularly peel extracts, have shown strong antioxidant activity [17]. Betalines, the predominant beta-cyanine in red beetroot, are mainly dispersed to the outer parts of the root, declining in the order of peel, crown, and flesh. Betaline and isobetaline exist in the peels in larger concentrations than in the beetroot tissue Kujala et al. [18]. Beetroot peel has nutraceutical potential, and has an ability to scavenge free radicals and inhibit microorganism activity [16].

Moreover, beetroot peels are high in dietary fibers, minerals and also have 50% of phenolics, whereas the flesh contains only 13% [19], representing a potential for beetroot by-products to be more accessible to use in food products. A previous study has attempted to use beetroot as a preservative to extend the shelf-life of various foods, such as mayonnaise and dairy products [20,21]. At the same time, only one study has aimed at using beetroot as an antioxidant in the preservation of seafood, such as Deccan Mahseer [22].

The current study aimed to evaluate the extent of secondary plant metabolites (phytochemicals) and establish the antioxidant activity of dried red beetroot peel (DBRB) extract. Additionally, we aimed to compare the quality characteristics and shelf-life of raw Nile *Tilapia* fillets pretreated by dipping in aqueous DBRP extract vs. BHT before refrigeration. Monitoring chemical characteristics such as TBA and sensory changes after cold storage at 5 ± 1 °C for 10 days indicated the preservation effect.

## 2. Materials and Methods

### 2.1. Material and Chemicals

Fresh red beetroot and Nile *Tilapia* fish fillets were purchased from a local market, Giza, Egypt. 2,2-diphenyl-1-picryl hydrazyl, (DPPH) Folin–Ciocalteu, aluminum chloride, gallic acid standard, hydrochloric acid, BHT, thiobibatioric acid (TBA) and trichloroacetic acid were purchased from Sigma Aldrich Chemical Co., St. Louis, MO, USA.

### 2.2. Drying of Red Beetroot Peels

Red beetroot roots were washed thoroughly under tap water. The peels were removed using a sharp sterile knife, then sliced into small thin slices and placed in the oven at 65 °C for 7 h to dry. The dried peels were ground to powder in a blender.

### 2.3. Preparation of Dried Red Beetroot Peels Aqueous Extract

Extraction was performed using the maceration technique of peel powder (5 g/100 mL distilled water), the extract was kept in the shaker (300 rpm) for 1 h, then the extract was filtrated using filter paper (Watman No.1). All extracts were analyzed in triplicate.

### 2.4. Estimation of Total Phenol Content

Total phenolic content was estimated by the Folin–Ciocalteu reagent method according to Abdelgawad et al. [23] with some modifications. The DBRP extract solution (0.1 mL) was mixed with 1 mL of Folin–Ciocalteu reagent (1:10 diluted with distilled water), and after 5 min, 1 mL of 7.5% Na_2_CO_3_ solution was added. The mixture was incubated in dark at room temperature for 1 h. The A was measured at 760 nm by spectrophotometer (model UV-2401 PC, Shimadzu, Milano, Italia). The total phenolic content was assessed in terms of gallic acid equivalent mg/100 g of dried peels.

### 2.5. Estimation of Total Flavonoids Content (TFC)

The total flavonoids content (TFC) was carried out using the method of Mohamed et al. [24] with some modifications. The total flavonoid content of DRBP extract was analyzed using catechin as standard and expressed as mg catechin/100 g DRBP. In brief, 0.25 mL of the previous extract was added to 0.75 mL of methanol and then 50 μL of aluminum chloride 10% and 50 μL of potassium acetate were added. The mixture was completed to 1.40 mL with distilled water and kept for 30 min in the dark. The absorbance was measured at 430 nm.

### 2.6. Phenols and Flavonoids Characterization

The phenolic components and flavonoids in the DBRP extract solution were identified using high-performance liquid chromatography (HPLC). HPLC analysis was performed using an Agilent 1260 Infinity HPLC series (Agilent Technologies, Santa Clara, CA, USA) equipped with a quaternary pump and a Zorbax Eclipse plus C180 column (Agilent Technologies, Santa Clara, CA, USA) with a 100 4.6 mm i.d. A triple linear elution gradient was used to separate the samples: (A) HPLC grade water 0.2 percent H_3_PO_4_ (*v*/*v*), (B) methanol, and (C) acetonitrile. The volume injected was 20 μL. For estimated phenolic acids, all chromatograms were plotted at 284 nm, and for flavonoids, at 330 nm. By comparing peak areas to external standards, all components were recognized and quantified.

### 2.7. Estimation of Betalain Content

The content of betalains in the extract was calculated as g/100 g dry DRBP. The extraction was measured at wavelength 535 nm. The quantification of betalains was calculated according to Singh et al. [25] using the following Equation (1):

Betalain content = A × DF × MW × 1000/ϵ L (1)

where:

A: the value of absorption at 535 nm, DF: Dilution factor, L: Cuvette’s light path length (1 cm), MW: Betaline’s molecular weight (550 g/mol). The betalain’s extinction coefficient (60,000 L/mol).

### 2.8. Estimation of Radical-Scavenging Activity by (DPPH IC50%)

The IC50% was measured using the DPPH assay according to Ali and El Sayed [26]. The DBRP extract (0.1 mL) was taken in different concentrations (50, 80, 100, and 200 mg/100 mL) and mixed with 3.9 mL of DPPH solution (0.0024 mg/100 methanol). The mixture was kept in dark at room temperature and the absorption was measured at 517 nm after 30 min using a spectrophotometer. The free radical scavenging was expressed as % of inhibition according to Equation (2):

(2) $$\mathrm{Inhibition}\;\left(\%\right)=\frac{A_{\mathrm{control}}-A_{\mathrm{sample}}}{A_{\mathrm{control}}}\times 100$$

where A control and A sample are the absorbance of the control and sample, respectively.

### 2.9. Preparation of Fish Filets

The fish fillet pieces were cut into equal small cubes weighing 25 g, 7 × 7 cm, and 5 cm in thickness. Four different treatments were used for preserving fish fillets, as presented in Table 1.

All treatments were dipped into their respective concentration solutions for 5 min. The samples were screened of the excess dipping solution and dried at room temperature, then packed in polyethylene (LDPE) pages with thickness of 80 μm, water vapor permeability of 44.756 ± 0.12 g mm m^−2^ day^−1^, oxygen permeability of 5.42 ± 0.18 cc m^−2^ day atm and length × width (15 × 10 cm) and afterwards stored at 5 °C for 10 days. The samples were drawn randomly in triplicates at 3-day intervals for chemical and sensory evaluations.

### 2.10. Determination of TBA and pH

Thiobarbituric acid (TBA) was determined according to Abdel Aziz et al. [27]. Minced fish sample (5 g) was homogenized for 1 min in 20 mL of trichloroacetic acid (TCA solution 10% *w*/*v*). Five ml of the extract solution and 5 mL of the TBA reagent (0.02 M) were transferred and blended well into a glass tube. In a 20 min water bath, the tube was heated to 90 °C. After cooling, the absorbance was measured at 530 nm using a spectrophotometer (Unico UV-2000, UNICO company, Fairfield, NJ, USA). A standard curve of 1,1,3,3-tetra methoxy-propane as a substrate for malondialdehyde was used to measure the TBA value) mg malondialdehyde MDA kg fish tissue. For pH determination, the fish fillet sample (5 g) was homogenized for 2 min in 50 mL distilled water. Two layers of cheesecloth were used to filter the homogenate. The pH meter (Jenway, layout 3305, Dunmow, Essex, UK) was used to assess the filtrate’s pH.

### 2.11. Sensory Evaluation

According to Abdel Aziz et al. [27], sensory evaluation for color, odor, texture and overall acceptability of raw uncooked as well as the flavor of cooked fish fillet samples was conducted (the grill was preheated to 230 °C after brushing with olive oil). For cooking, fillets were placed on the grill and cooked until the internal temperature of the fish reached 65 °C, and fillets were flipped as needed to prevent either side from burning and were evaluated on the same day. Additionally, through the cold storage, only raw uncooked fish fillet samples were rated. Thirty panelists of the Department of Food Science, Faculty of Agriculture, Cairo University’s members (20 females and 10 males, their ages ranging from 25 to 40 years) presented scoring using a 9-point hedonic scale (0–2 = dislike extremely, 3–4 = dislike slightly, 5 = fair, 6–8 = like moderately, and 9 = excellent). The rejection limit was 4, which is considered the quality borderline of consumer acceptance.

### 2.12. Statistical Analysis

All statistical analysis was conducted using CoStat Version 6.45 (CoHort Software, Monterey, CA, USA). Primary statistical analysis was one-way analysis of variance (ANOVA). Normality distributions in each experiment were checked using the Shapiro–Wilk test. Duncan’s multiple range test was conducted at a significant point of 5%. Bartlett’s test was used to test the homogeneity of variance between sample groups. SigmaPlot Version 12 (Systat Software Inc., Erkrath, Germany) was used to create graphs and figures.

## 3. Results and Discussion

### 3.1. Phytochemical Profile of DBRP

The total polyphenols were determined by the Folin–Ciocalteu assay, which are the dominant bioactive compounds (832 mg/100 g) in the DRBP. Moreover, flavonoids represent almost 25% (234 mg/100 g) of the total polyphenols present in the DRBP. Meanwhile, the betalains content reached 535 mg/100 g. These obtained results were higher than those assessed by Tumbas Šaponjac et al. [28], which showed the contents of total polyphenols (326.51 mg GAE/100 g), flavonoids (10.23 mg RE/100 g), and betalains (60.52 mg betanin/100 g and 61.33 mg vulgaxanthin-I/100 g) in aqueous ethanol extract of dried beetroot pomace. Additionally, the obtained results were less than that assessed by Lazar et al. [19], who showed that beetroot peel powder water extract had a high betalain content (1.18 mg/g DW) and rich polyphenolic content (225.36 mg GAE/g DW). Vuli’c et al. [29] varieties evaluated beetroot pomaces such as Cardeal-F1, Egyptian, Bicor, and Kestrel. They reported that the TPC in beetroot pomaces varied from 1.87 to 11.98 mg/g DW and betalain content ranged from 0.75 (for cv. Egyptian) to 3.75 mg/g DW (for cv. Bicor). Finally, compared to the literature data presented here, our results show higher values of total polyphenols and betalains, which is because we worked with DBRP extract. Kujala et al. [18] found that the TPC of red beetroot increases in the following order: flesh (13%), crown (37%), and peel (50%).

### 3.2. Identification and Quantification of Phenolic Compounds and Flavonoids in DBRP by HPLC

Table 2 identifies the phenolic compounds and flavonoids and their percentages for DBRP extract. The highest phenolic compounds which were identified for DBRP extract were syringic acid (55.01 mg/g), chlorogenic (38.78 mg/g), catechol (24.98 mg/g), p-coumaric acid (13.12 mg/g), ferulic acid (2.45 mg/g), o-coumaric acid (2.4 mg/g), and iso-ferulic acid (1.97 mg/g), respectively. In accordance with our results, previous works recorded some phenolic compounds for DBRP extract, including p-coumaric acid, o-coumaric acid, syringic acid, and ferulic acid [22,30,31]. The predominant phenolic acids such as gentisic, ferulic, and p-coumaric acid have moderately antioxidant activity due to their structural elements [32]. The high antioxidant activity of DBRE is related to the existence of major phenolic compounds such as syringic acid, chlorogenic, catechol, p-coumaric acid, and ferulic acid, which were detected in this study and previous work [33]. Chlorogenic acid is a natural phenolic compound found in various plant species and is also one of the main phenolic components of DBRP extract. According to common perception, chlorogenic acid and similar chemicals are recognized as antioxidants. Lan [34] stated that the extent of chlorogenic acid in *Flos Lonicerae* extracts reflects the antioxidant activity. The current findings show that a larger chlorogenic acid concentration increases the efficacy of scavenging the DPPH radical. On the other hand, several flavonoid compounds were found in the extract of DBRP such as rutin, naringenin, rosmarinic, hesperdin, quercetin, kaempferol, catechein, myricetin, apignin, and hespertin (Table 2). Naringenin (0.146 mg/g), rutin (0.090 mg/g), and hesperdin (0.040 mg/g) were found in high quantities in DBRE extract. This result does not agree with Maqbool et al. [22], who found that in beetroot powder extract, rutin and hesperdin levels were lower than expected.

### 3.3. DPPH Radical-Scavenging Capacity of DBRP

**Figure 1:**
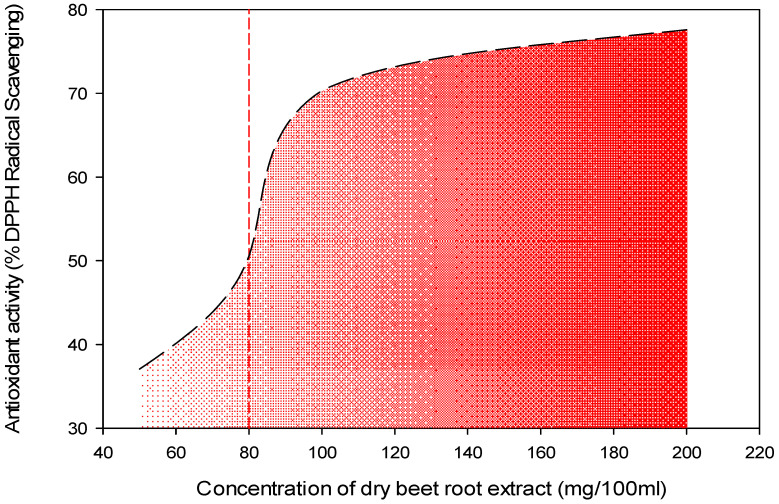
Antioxidant activity % of an IC50 at 80 mg/100 mL of DBRE (red dotted vertical line) dry beetroot extract.

Figure 1 showed a DPPH radical-scavenging capacity ranging from 37.06 to 77.62% at different concentrations of DRBP, beginning from 50 mg/100 mL to 200 mg/100 mL DRBP methanolic extract. Additionally, the IC50% was recorded at 80 mg/100 mL DRBP extract. Meanwhile, Maqbool et al. [22] reported that the radical-scavenging activity ranged from 70.12 to 0.90% for high concentrations (4–20%) of beetroot peel extract. It was noticed that the scavenging activity of DBRP increased with an increase in extract concentration. Our results agree with Karimi et al. [35], showing a linear correlation between phenolic content and antioxidant activity. Additionally, flavonoids are a ubiquitous group of phenolic compounds responsible for in vitro antioxidant activity, primarily because of derivatives of benzo-γ-pyran [36]. Their role as an antioxidant effect is to chelate metal [37]. Beetroot’s antioxidant activity is attributed mainly to amino and hydroxyl groups and phenolic compounds [38]. Therefore, red beetroot has been ranked as one of the ten leading vegetables with superior antioxidant activity [30]. Deliorman Orhan et al. [39] reported that the location, genotype, cultivation techniques, and differences in the plant’s maturity affect the content of phenolic compounds. Additionally, the type of solvent, extraction methods and conditions, and measurement methods applied can affect the content and composition of bioactive compounds such as phenolic compounds [19]. The water extract was preferred for food preservation. Indeed, using water as a solvent to extract phenolic compounds depends on natural material that can be nontoxic, safe, and economical [24].

### 3.4. Change in pH Values of Nile Tilapia Fish Fillet

**Figure 2:**
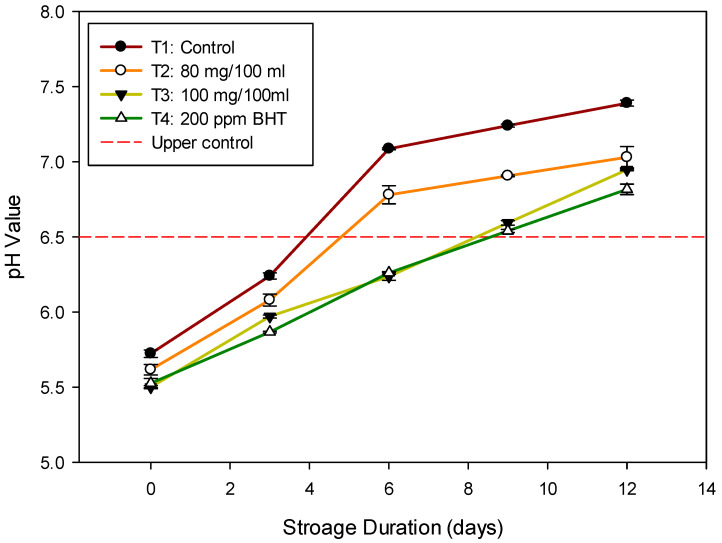
pH values of Nile *Talipia* fillets treated with DRBP during storage for 12 days at 5 °C.

The *Tilapia* fillet’s initial pH values ranged from 5.49 to 5.72, which indicates the freshness of the fish. The changes in pH values during storage at 5 °C for 12 days are shown in Figure 2, which were slightly different from previous studies that showed an initial pH of 6.2 [10]. The initial pH values differ from those reported in other studies due to the species, catching season, diet, stress level during the catch, and muscle type [40]. As the quality degrades, the increase in pH was observed primarily due to the production of alkaline bacterial metabolites and the increase in total volatile basic nitrogen levels [41]. In the current study, the lowest pH level was observed for T4 (200 ppm BHT) followed by T3 (100 mg/100 mL of beetroot extract). The pH values were significantly higher (*p*-value < 0.05) between treatments from the 2 days of storage onwards, and which gradually increased for control samples (T1) above 7 as shown in Figure 2. According to EOS (2005) [42], the maximum acceptable pH limit is 6.5 in fish and fish products. In the current study, the pH values of T3 and T4 did not rise above 7 throughout the cold storage for 12 days, which is in agreement with Maqbool et al. [22] who reported that the frozen Deccan mahseer fish samples treated with beetroot peel exhibited a lower pH value of 6.73 than the control sample that exceeded the acceptability limit of 7.08. These results indicate that using either 100 mg/100 mL beetroot extract or 200 ppm BHT kept the pH of fish samples significantly lower than control samples during a 12-day storage period. A rise in pH implies bacterial growth, deterioration in quality, and the possibility of spoiling [43]. pH is a good indicator of the freshness of fish and fish-based products, as it starts low at the beginning of storage and rises gradually during the storage period [44].

### 3.5. Changes in Thiobarbituric Acid (TBA) Value of Nile Tilapia Fish Fillet

**Figure 3:**
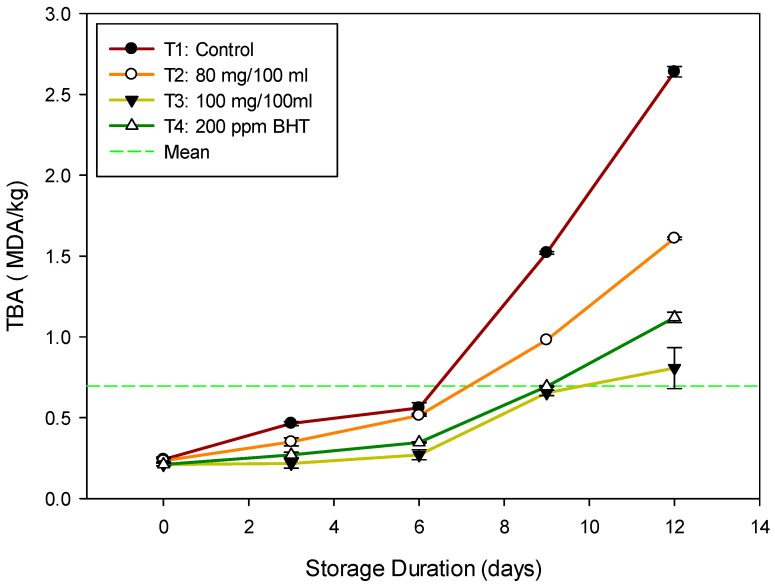
Mean TBA value, with standard deviation bars of Nile *Talipia* fillets during storage for 12 days at 5 °C.

TBA is a lipid oxidation index that measures malondialdehyde (MDA) concentration. MDA comprises hydroperoxides, the first products of polyunsaturated fatty acids reacting with oxygen [45]. TBA values for the treated fish samples are presented in Figure 3. TBA levels were kept constant (~0.5 mg of MDA/kg) for the *Talipia* fillets for all the treatments for up to six days of storage. A sharp increase in TBA value was observed from six days onward for all the treatments. However, the increase was significantly lower for T3 (0.8 MDA/kg *Talipia* fillets) after 12 days of storage. The control sample was 2.6 mg of MDA/kg *Talipia* fillets by the end of the storage period. All treatments did not exceed the acceptability limit (4.5) as per the guidance of EOS [42]. The breakdown of hydroperoxide into secondary oxidation products, and major aldehydes at a later stage of lipid oxidation, could explain the increase in TBA level in the control sample [46]. The reduced TBA value in treated fish fillets could be due to various mechanisms, including the inhibition of radical chain initiation, peroxide breakdown, and free radical interaction [47]. The obtained results agree with Maqbool et al. [22] who reported that the frozen Deccan mahseer fish samples treated with beetroot peel exhibited a lower TBA (1.5 MDA/kg *Talipia* fillets) after 180 days of storage compared to the control which reached 2.5 MDA/kg.

Moreover, the findings are consistent with Mohamed et al. [24], who reported that eel fillets coated with CMC + 1.5% of oregano essential oil had the lowest TBA value (0.65 mg MDA/kg eel fillets). The reduced increase in the treated fish samples with DBRP compared with control possibly was due to the antioxidant activity of DRBP, as mentioned before. The DRBP extract contains phenolic acids such as ferulic, chlorogenic, p-coumaric, p-hydroxybenzoic, and syringic acids, as well as betalines and flavonoids with antioxidant properties, particularly chlorogenic acid.

### 3.6. Changes in Sensory Analysis of Nile Tilapia Fish Fillet

**Figure 4:**
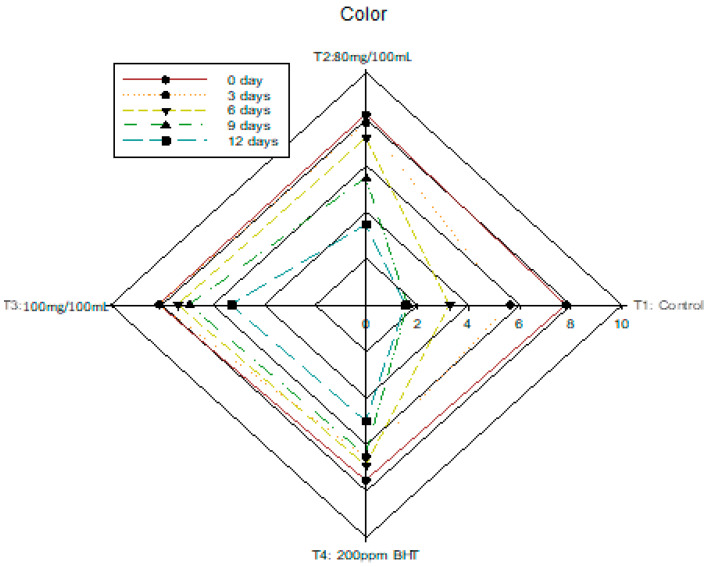

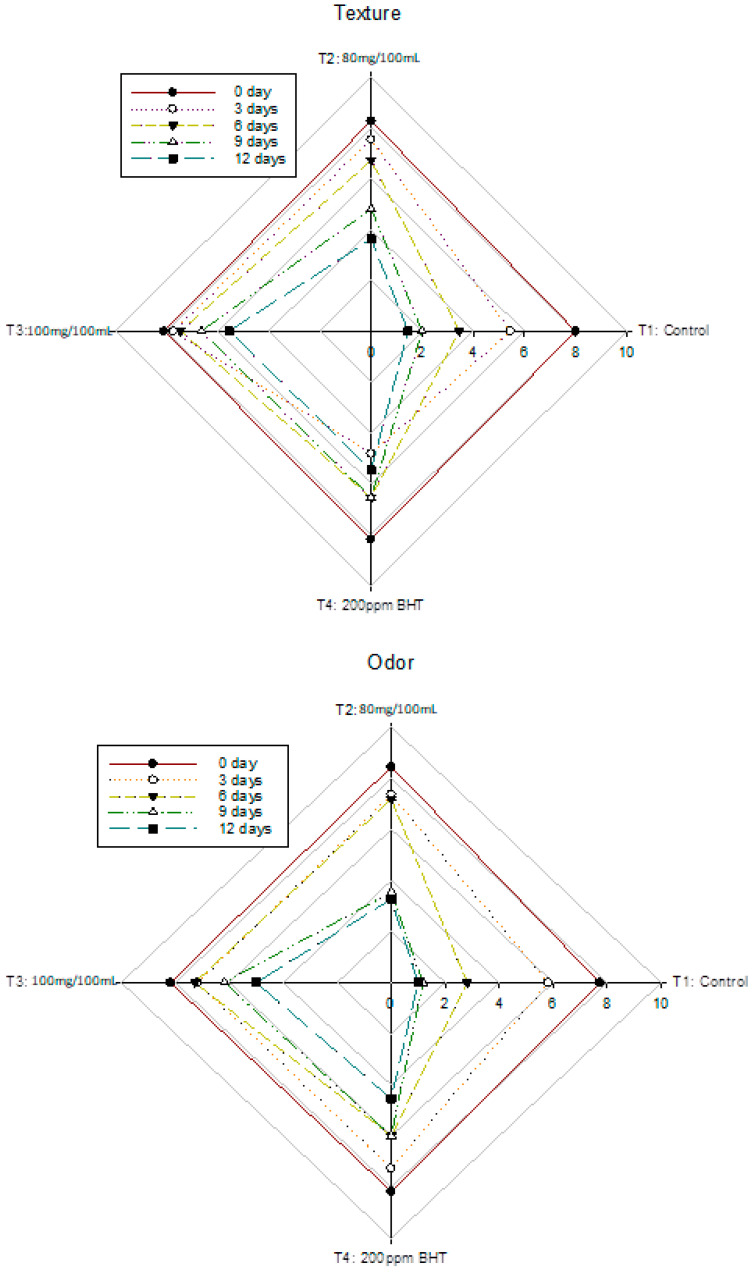

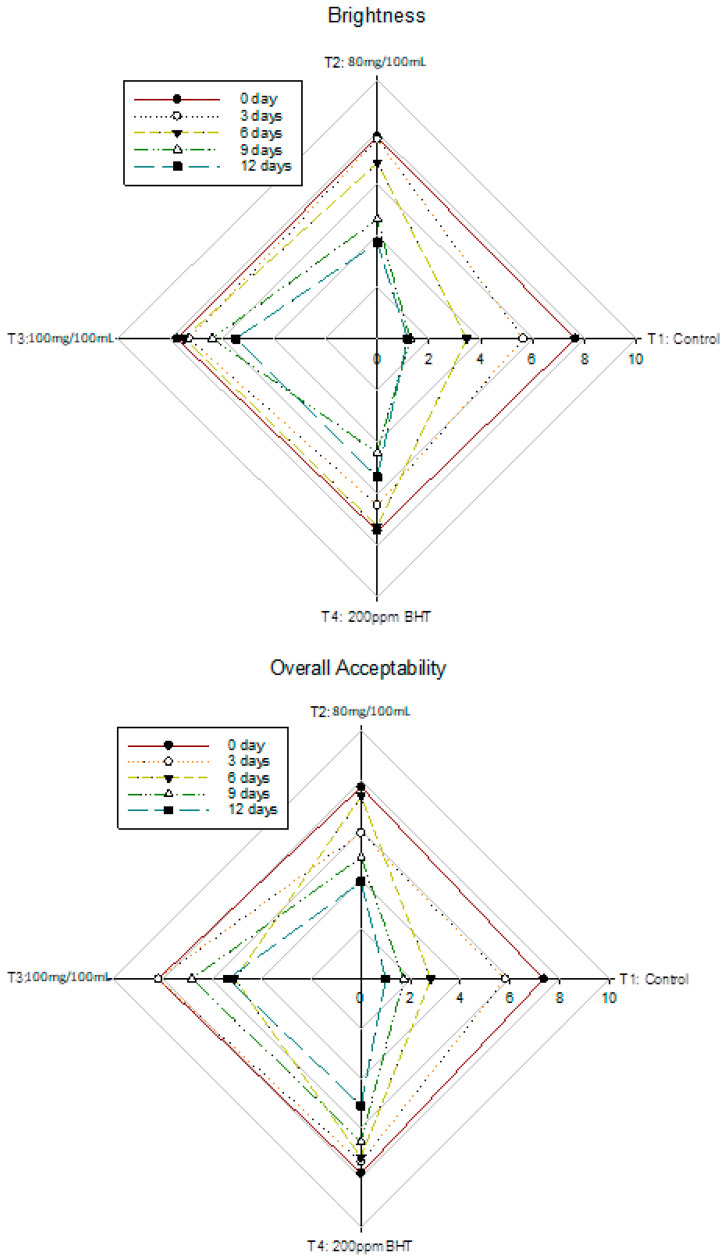
Changes in sensory parameter scores of *Nile Talipia* fillets during storage at 5 °C for 12 days.

All the sensory parameters of the fresh and cooked fish fillets were rated on the first day of storage. The sensory qualities of *Tilapia* fillets were not affected by dipping them in beetroot peel extract, especially flavor. The sensory parameters of uncooked fish fillets showed an inverse relationship to the storage days, with a gradual decrease in the score as the storage days progressed. Figure 4 presents the mean scores for the color and texture sensory analysis. The results indicated that T3 (100 mg/100 mL DRBP extract) and T4 (200 ppm BHT) result in the most acceptable quality by the end of the storage period (12 days) with scores ranging from 5 to 6 for color and texture, which did not reach the rejection limit (4). The ANOVA results showed the difference between samples treated with DRBP extract and BHT was significant (*p*-value < 0.05). For odor and brightness of the fish samples, T3 and T4 performed best compared to the control (Figure 4). They were still acceptable after 12 days of storage; meanwhile, the control sample was rejected after 6 days of cold storage. Rancidity was sensorially detected by the panelists on the 6th day of storage for T2, but the 3rd day for the control sample. That may be due to the DRBP’s content of TPC, especially chlorogenic acid [34], which made red beetroot rank as one of the ten leading vegetables with superior antioxidant activity [30].

All treated samples were acceptable after 12 days of storage. Moreover, T3 was the most acceptable; it had the highest overall acceptability and remained as good as fresh samples after 12 days of storage (Figure 4). This may have to do with active components, including carotenoids, glycine, and antioxidant compounds such as betaines, polyphenols, flavonoids, and isothiocyanates [16,48]. According to sensory results, non-treated *Tilapia* fillets had a shelf-life of three days, but treated samples had a shelf-life of more than nine days in samples treated with a low concentration (80 mg/100 mL water) of DBRP extract and more than twelve days in samples treated with a high concentration (100 mg/100 mL water) of DRBP. The sensory analysis results are consistent with the TBA value, indicating that the DRBP can limit oxidation during cold storage.

## 4. Conclusions

In this study, we evaluated the phytochemicals of dried red beetroot peel extract and its effect on the shelf-life of Nile *Talipia* fish fillet under cold storage conditions. The results indicated that red beetroot extract contains several phenolic compounds such as syringic acid, chlorogenic, catechol, p-coumaric acid, ferulic acid, o-coumaric acid, and iso-ferulic acid. Additionally, several flavonoid compounds were found in the extract such as rutin, naringenin, rosmarinic, hesperdin, quercetin, kaempferol, catechein, myricetin, apignin, and hespertin. As a result, the antioxidant activity of the extract was high. The potential of red beetroot as an antioxidant was revealed by determining the IC50%, which is the concentration required to produce 50% antioxidant activity (80 mg/100 mL DRBP extract). Subsequently, two concentrations for preserving fish fillets were chosen: the IC50% concentration and the 100 mg/100 mL DRBP extract. Treated Nile *Talipia* fish fillets resulted in lower pH and TBA and higher sensory parameters (without any negative effect on flavor on the initial day) during cold storage compared with control. Therefore, the optimal concentration of red beetroot extracts for preserving Nile *Talipia* fish or other types of fish with similar physiology is 100 mg/100 mL; this concentration yielded the same results as commercially used synthetic antioxidants such as BHT. It is concluded that such novel extracts can prove to be sustainable preservatives for fish and fish products and reduce waste along the agricultural supply chains.

## Figures and Tables

**Table 1:** Treatments and their concentrations.

Treatment	Details/Concentration
T1 (Control)	fish fillet cubes were dipped in distilled water
T2	fish fillet cubes were dipped in aquatic DRBR extract (80 mg powder/100 mL water)
T3	fish fillet cubes were dipped in an aquatic extract of DRBR (100 mg powder/100 mL water)
T4	fish fillet cubes were dipped in BHT solution (200 ppm).

**Table 2:** The quantity of the identified phenolic compounds and flavonoids (mg/g) in DBRE extract as estimated by HPLC.

Phenolic Compounds		Flavonoids	
Pyrogallol	0.151	Rutin	0.090
Quinol	-	Naringenin	0.146
Gallic	0.019	Rosmarinic	0.003
Protocatchoic	0.011	Hesperdin	0.040
Catechol	24.981	Quercetin	0.008
*P*-Hydroxy benzoic acid	0.018	Kaempferol	0.001
4-Aminobenzoic	0.003	Catechein	0.003
Salicylic acid	-	Myricetin	0.002
Chlorogenic	38.870	Apignin	0.0001
Caffeine	0.009	Hespertin	0.0002
Benzoic acid	0.012		
Caffeic acid	0.028		
Vanillic acid	-		
*P*-Coumaric acid	13.122		
Syringic acid	55.012		
Ferulic acid	2.450		
Iso-Ferulic acid	1.971		
*O*-Coumaric acid	2.042		
Coumarin	0.691		
Cinnamic acid	0.007		

## Data Availability

Data is contained within the article.
